# Strategies for Reducing Suicide at Railroads: A Scoping Review of Evidence and Gaps

**DOI:** 10.3390/ijerph22010018

**Published:** 2024-12-27

**Authors:** Pooja Belur, Patrick Sherry, Ivan Rodriguez, Chetan Kurkure, Shashank V. Joshi

**Affiliations:** 1 Department of Psychiatry, Keck School of Medicine, University of Southern California, Los Angeles, CA 90033, USA; 2 University College, University of Denver, Denver, CO 80208, USA; patrick.sherry@du.edu; 3 Department of Psychiatry and Behavioral Sciences, Division of Child and Adolescent Psychiatry and Child Development, Stanford University School of Medicine, Stanford, CA 94305, USA; ivan3@stanford.edu (I.R.); ckurkure@stanford.edu (C.K.); svjoshi@stanford.edu (S.V.J.)

**Keywords:** suicide prevention, means restriction, railroads, systematic review, physical barriers, media reporting

## Abstract

This review aims to systematically evaluate existing literature on reducing suicides along railroads, with specific focus on effectiveness, limitations, and research gaps in the current evidence base. Database searches were conducted in PubMed, PsycInfo, Scopus, Embase, and CINAHL covering studies published until 30 November 2024. After screening 623 studies and their references, 51 studies were included; 26 empirically assessed rail-related prevention interventions and 25 provided relevant qualitative insights. Physical barriers like removal of grade crossings, addition of fencing, and platform screen doors (PSDs) showed significant promise. Full-height PSDs eliminated all suicides and half-height PSDs significantly reduced suicide incidence. Fencing was found to be effective but raised concerns about feasibility and must be part of a comprehensive approach to mitigate potential displacement. Safe media reporting was linked to decreased suicides and a reduced risk of contagion, and CCTV monitoring and suicide pits also showed potential but had limited research. Other strategies showed mixed evidence and required additional evaluation. Some studies, particularly on physical barriers, showed possible displacement effects to other stations, highlighting the need for studies larger in geographic and temporal scope. Our findings support certain prevention interventions, but generalizability is limited by scope of research and methodological concerns. Overall, our findings highlight the need for broader, long-term studies to confirm efficacy and establish comprehensive, scalable approaches for policy implementation.

## 1. Introduction

### 1.1. Background

Suicide is a significant public health concern, with profound impacts on individuals, families, and entire communities worldwide. In 2022 alone, over 700,000 individuals died by suicide, with countless more attempts recorded for each death [1]. Adolescents and young adults are disproportionately affected; suicide ranks as the second leading cause of death among 10–24-year-olds [1,2]. Consequently, suicide prevention has become a top priority for public health agencies and mental health practitioners worldwide [1].

Although railway suicides represent only about 1% of total suicides, they pose unique challenges due to the fact that they are difficult to prevent, and their lethality is significant [3]. The consequences not only affect the individuals directly involved but have considerable secondary effects as they also impact emergency responders, commuters, and the broader community. Train drivers in particular can be profoundly affected, as studies have shown increased rates of somatic symptoms, anxiety, sleep disruption, and post-traumatic stress disorder among train drivers witnessing or involved in such incidents [4,5,6]. Such psychological burdens harm the well-being of these professionals and compromise railway operations’ safety and efficiency [7]. Moreover, the indirect costs, including lost time, delays, insurance expenses, and legal proceedings, add to the economic and logistical toll of railway suicides [2,6,8,9,10].

Means restriction (MR) has emerged as a critical component of prevention strategies and interventions aimed at reducing access to methods commonly used in suicides. MR relies on the theory that removing or reducing access to lethal means can prevent suicide attempts or delay action for long enough to allow for intervention [11,12]. Traditional MR methods focus on restricting direct access to risk factors such as firearms, medications, and hotspots. MR strategies typically refer to physical barriers (e.g., fencing, platform screen doors). In the context of railway suicides, both means restriction and additional interventions may be needed.

This review focuses specifically on intervention strategies relevant to railways, noting that that while physical interventions are critical, non-physical approaches are equally important in developing comprehensive prevention strategies.

Looking beyond traditional physical interventions is essential in this setting, as it allows for a more comprehensive understanding of strategies that consider the unique contexts of railway systems. Therefore, our review provides further clarification on the factors affecting successful implementation and effectiveness in reducing suicide risk across a broader range of settings [11].

### 1.2. Scope

While research on railway suicides has increased, existing reviews have primarily focused on specific interventions or geographic regions without synthesizing the broader range of strategies across contexts [13,14,15,16,17,18]. This scoping review aims to fill this gap by offering a more holistic synthesis of both physical and non-physical approaches, exploring their context-specific effectiveness, and addressing a critical gap in the literature regarding how these strategies function across various settings and populations. Specifically, we aim to:

1. Identify and categorize prevention strategies currently implemented;
2. Assess the effectiveness of prevention strategies in reducing suicide rates attempts, and lethality of suicidal behaviors;
3. Explore key challenges, limitations, and unintended consequences associated with the implementation of prevention strategies along railroads;
4. Identify gaps in the current evidence and highlight areas for future research, practice, and policy development in suicide prevention.

## 2. Materials and Methods

**Figure 1:**
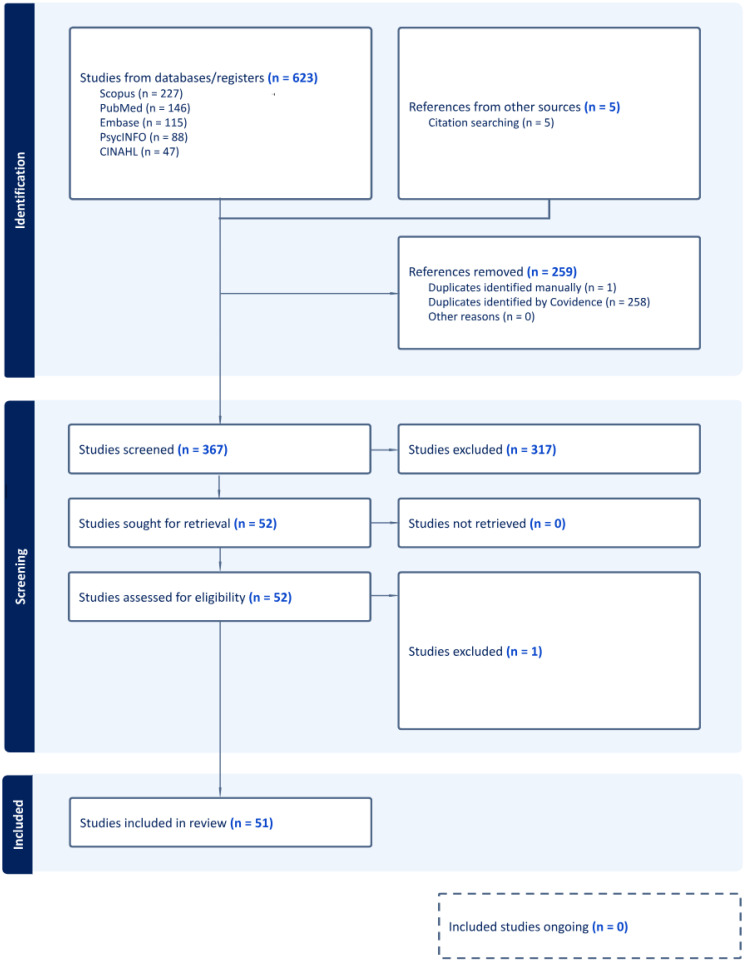
PRISMA flowchart illustrating the identification and screening process for the literature review on prevention strategies related to railways.

The review was conducted adhering to Preferred Reporting Items for Systematic Reviews and Meta-Analyses 2020 guidelines for systematic reviews and meta-analyses. The PRISMA flow diagram illustrates our process of study selection and data synthesis (Figure 1) [19].

### 2.1. Search Strategy and Data Sources

A comprehensive search was developed for English-language, peer-reviewed papers published before 30 November 2024, employing the following search terms: suicide, prevention, risk reduction, means prevention, means restriction, train, rail, railway, and railroad. Search terms were tailored to each database’s indexing to maximize relevant study retrieval. The search was conducted in PubMed, PsycInfo, Scopus, Embase, and CINAHL, with no restrictions on publication date. The bibliographies of retrieved papers from these databases were manually reviewed for pertinent citations.

### 2.2. Inclusion and Exclusion Criteria

Initial screening of abstracts and manuscripts was conducted independently by three independent authors (PB, IR, CK) using predefined inclusion and exclusion criteria. Cases of disagreement were resolved through discussion with a fourth independent author (SVJ). Inclusion criteria included: abstracts and full-length papers written in English or with accessible translations, studies addressing prevention strategies specifically related to train tracks, a focus on interventions, policies, and research aimed at preventing railway suicides. Exclusion criteria included: studies that did not discuss prevention strategies directly related to train tracks, non-empirical literature such as opinion pieces and editorials lacking empirical evidence, studies published after 30 November 2024, and those that were not available in English or could not be accessed through institutional resources.

### 2.3. Data Extraction

Data collection and extraction were also completed using Covidence [20]. A total of 623 studies were originally imported, which was reduced to 364 studies after duplicates were removed. This was reduced further to 47 following dual screening of the paper abstracts and titles. Five studies were added based on reference lists. One study, focused on grade crossings, was excluded after full-text retrieval due to limited empirical data directly relevant to prevention strategies (Figure 1). Data from these 51 papers were extracted using a standardized template to ensure consistency and comparability across studies. The following data were gathered for each study: author(s), publication year, location and time period, study design, prevention strategy used, evaluation metrics/main findings, and reported limitations.

### 2.4. Data Synthesis

Data synthesis was performed using a narrative approach due to the heterogeneity in intervention types, study designs, and outcome measures. Meta-analysis was deemed inappropriate due to the limited number of comparable studies and methodological differences among included studies.

### 2.5. Ethical Approval

IRB approval was not required for this literature review, as it involved the analysis of publicly available data from previously published studies.

## 3. Results

### 3.1. Descriptive Statistics

Our review identified 51 publications pertinent to prevention interventions at railroads. Twenty-six of the papers comprised epidemiological studies or purely theoretical assessments of interventions [2,3,6,9,21,22,23,24,25,26]. Twenty-four of the papers sampled cross-sections of suicides across a small number of test stations or sites [27,28,29,30,31,32,33,34,35,36,37,38,39,40,41,42,43,44,45,46,47,48,49,50]. One paper, by Agarwal, utilized globally acquired CCTV footage [51]. Another paper evaluated signage across several US railroad lines and stations [52].

**Figure 2:**
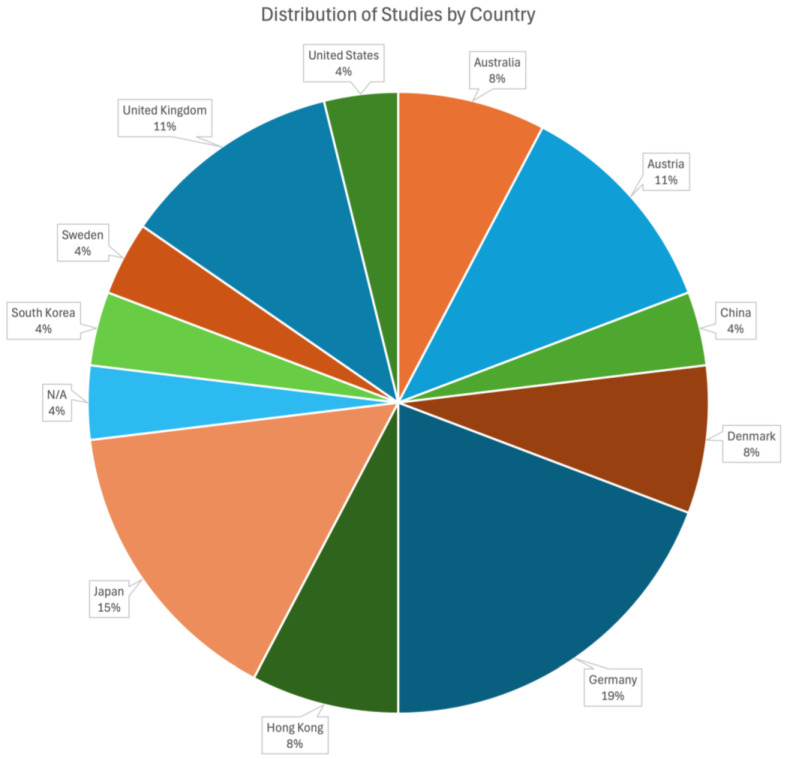
Distribution of 26 studies related to railways across ten countries.

**Figure 3:**
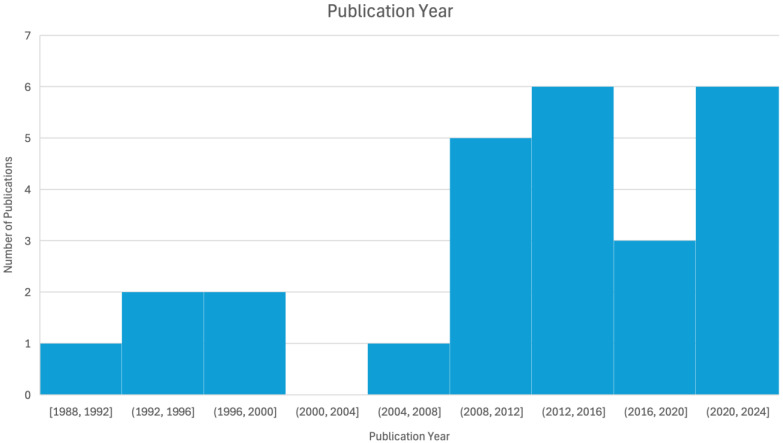
Histogram of publication years for the 26 evaluated manuscripts on prevention strategies related to railways.

**Figure 4:**
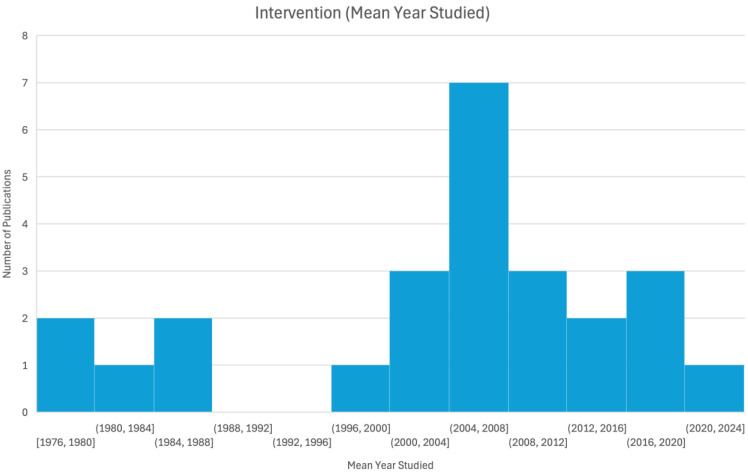
Histogram of average year of intervention implementation for 26 included studies.

Of the 51 identified studies, only 26 were included in our evaluation table as they contained empirical research that directly evaluated the effectiveness of interventions rather than purely theoretical discussion or broad epidemiological analyses (Table 1). Of these 26 papers, there were 15 unique interventions conducted across 11 countries, with Germany and Japan having the highest representation (Figure 2). Notably, certain interventions, such as those highlighted in German publications after professional footballer Robert Enke’s death in 2009, were overrepresented in the literature when considering the rate of suicides in Germany compared to the global average. The majority of studies were conducted post-2005, with an average publication year of 2011 (Figure 3). Interventions had a mean year of evaluation between 1982 and 2022, with a left skew indicating more interventions in recent years (Figure 4). Most studies focused on passengers at railway stations, particularly those who attempted or died by suicide. One exception, by Katsampa et al., focused on individuals who intervened during suicide attempts along the railroad right of way or on platforms [27]. Methodologies varied, with 21 studies being non-randomized, observational, and quasi-experimental. In these papers, data were either analyzed within site(s), before and after an intervention, or between nearby sites with and without the intervention. The remaining papers included four media analysis papers and one qualitative, interview-based report.

### 3.2. Interventions

Quantitative findings were sparse across all interventions, as empirical studies typically concentrated on specific train lines or city regions and assessed only a small number of suicides. A review by Havarneanu et al. identified three major categories of preventative activities and interventions: (1) deterrence (platform screen doors, physical barriers, calming blue light, appropriate media reporting); (2) detection (monitoring and detection system, surveillance unit); and (3) response (pits between rails, staff training to approach people) [2,6,13,14,15,16,17,53,54]. The heterogeneity in methodologies, geographic constraints, and small sample sizes in many studies rendered quantitative data synthesis through meta-analysis unfeasible. As a result, we summarize the effectiveness of interventions qualitatively. Additionally, no subgroup or sensitivity analyses were conducted due to the diverse nature of the interventions and the limited data available across studies.

#### 3.2.1. Structural Deterrence and Limiting Access

Physical barriers have garnered substantial support as a powerful means of deterrence, though some concerns exist surrounding financial tenability [6,13,14,55]. The question of substitution or displacement to other locations is unresolved. Some research exists to suggest that displacement and substitution do not necessarily occur when individuals are prevented from using one particular method. However, systematic data are still lacking.

##### Fencing

Fencing is commonly employed to directly limit access to railroads, whether at stations or along track lines. The effects of introducing fencing are rarely studied as most assume the results are self-evident. Various materials and heights have been proposed for fencing [55]. In the case of rail suicide, a displacement effect may occur where those determined to take their own lives seek alternative access to the rail line. Three empirical studies investigated the effects of fencing. In New South Wales, RailCorp installed plain top chain-link fences in urban areas to reduce suicides and accidents. Gregor et al. reported 171 suicides and 842 attempts during a 7-year period, noting that fenced areas exhibited lower suicide rates overall compared to unfenced areas, and in particular, a lower proportion in non-jumping incidents (wandering, sitting, or lying on tracks) compared with jumping incidents (33% in fenced areas vs. 74% in unfenced areas) [40]. However, the actual type and extent of fencing was roughly 1800 mm in height and there was little detail as to how long sections were. Moreover, while the comparisons of the fenced versus unfenced areas is noted, the extent of fencing and the sections fenced are also not clear.

Additionally, the Swedish Transport Agency implemented 1-m-high mid-track fencing at one of seven stations along a single train line in 2013–2014. Despite a 62.5% decrease in suicides at the intervention station, there was a 162% increase at the control stations along the same line, indicating possible displacement to areas along the same train line [35]. This study provides pictures of the actual fencing, which are helpful; however, the barrier imposed could be easily breached or climbed over. It is hard to imagine that this is a serious deterrent if used uniformly across all stations.

More recently, Fredin-Knutzén et al. conducted an additional fencing study involving “platform end fencing” which found that PLF showed a large and significant reduction in trespassing from platform-ends, dropping from an average of 3.57 trespassers to 0.36 trespassers a month (*IRR* = 0.101). Additionally, a non-significant reduction from 1.11 to zero person under train events (PUTs) per year (*IRR* = 0.32) occurred. The number of PUTs also declined across the three control groups, suggesting that the preventive effect was not due to wider societal events affecting all stations. This study is commendable for the use of well-defined fencing descriptions, as well as the use of three different control group comparisons. The control group data were able to account for several aspects: similar traffic intensity, geographic areas, and similar station accessibility to the tracks. Lastly, the metrics used allowed for measurement of PUT (suicide) incidents, trespassers as well as train delay minutes. Train delay minutes allows a direct measure of the impact on operations and the riders [50].

Several other countries have begun fencing projects [8,55]. Communities in the U.S. (Palo Alto, California), Sweden, and France have built fencing between 6 to 10 feet in height. Most also incorporate upward or outward extensions at the top of fences, designed to make it more difficult to climb over, thereby further discouraging scaling over the fences [56,57]. However, there is still no empirically validated, standardized height for such fencing [55]. Several other countries have initiated fencing projects as well. Furthermore, the effectiveness of these barriers is influenced by various other factors, including length, coverage around the track, and materials used.

Methodological considerations for supplying exact dimensions, percentage of railway covered, extensive data on local suicide rate as well as numbers of attempts and deaths are needed to fully assess the impact of these barriers.

##### Platform Screen Doors (PSDs)

Studies investigating PSDs have been conducted in Hong Kong, Tokyo, Seoul, and Shanghai [30,34,36,37,41]. Data collected between 1997 and 2007 show that, following installation of PSDs, Hong Kong observed a significant reduction of 59.9% in the 5-year-average annual number of suicides, without significant displacement to unsealed platforms [36]. One study concluded that installation costs of HKD 2 billion were less than the savings obtained from reduced fatal suicides by 78.9%, improved Disability Adjusted Life Years (DALYs) by over 75%, and the cost of prevented service disruptions by 69.4% [41]. Additionally, studies suggest PSDs improve air quality, energy consumption, safety measures, and platform cleaning costs [58]. Japan was the first country to publish studies comparing half- and full-height PSDs. Overall, there was a 76% decrease in suicides following installation of PSDs. All seven suicides following installation occurred at stations with half-height PSDs, and five occurred because individuals climbed over the barrier. That said, costs were lower, and installation was easier for half-height PSDs [36].

Similar findings were noted in Seoul with a significant decrease of 89% in fatal cases. The three total suicides that occurred were at stations with half-height (65 inch) PSDs [37]. Similarly, Xing et al. investigated suicide rates following the installation of three types of PSDs. Overall, suicides declined by 90.9% with non-significant displacement to control stations. Suicide reductions were: 100% with full-height, 79.2% with 1.5 m high, and 60.2% with 1.2 m high [30]. Analyses of suicide rates from comparison sites did not support the presence of displacement or substitution effects. By the conclusion of the study period, all platforms in the system studied were using PSDs. Thus, studies point to the considerable effectiveness of full-height PSDs and the need to consider the cost benefits of half height or higher PSDs as the deterrent of choice.

##### Suicide Pits

“Suicide pits”, also referred to as drainage pits, are shallow trenches commonly installed between the rails to increase the distance between individuals and oncoming trains, potentially reducing the severity of injuries. These pits were not initially designed as suicide prevention measures but were already present in some rail systems, particularly in the London Underground [43].

Two studies examined the impact of 1-m-deep suicide pits on suicide and injury outcomes, and they found a 21% lower suicide mortality rate and a 32% lower overall mortality rate at stations with these pits compared to other stations [43,44]. However, it should be noted that these findings were comparisons rather than direct evidence of reduction due to the pits, as there were no baseline data available for comparison. Additionally, the studies did not account for differences between control and intervention stations, such as train speeds, passenger volume, and train frequency, which may have influenced the results.

#### 3.2.2. Detection and Monitoring Systems

##### Camera Detection

Closed-circuit television (CCTV) systems are recommended in railway settings to enhance safety by monitoring platforms and surrounding areas [16,53,59,60]. Various mechanisms of identification and detection using CCTV footage have been proposed to optimize sensitivity and specificity while minimizing false alerts [60]. In 2021, Agarwal proposed an automated method to detect at-risk individuals based on 11 patterns and visual features, including high-risk behaviors, gender, age, and emotional recognition at stations. The study identified 28 recordings with and 24 recordings without attempts. The system demonstrated a high sensitivity of 92.85% in detecting potential suicide attempts, although authors noted findings were limited by poor CCTV footage quality and short video durations [51].

In the US, Caltrain has tested camera detection and monitoring at four sites along their line, using visible and thermal infrared cameras to detect at-risk individuals on the tracks. The installation costs were USD 1.5 million, with USD 325,000 annually for maintenance and remote monitoring services. Empirical evidence as to the effectiveness of this system is not yet available [55].

##### In-Person Monitoring: Staff, Police, Security

Our review did not identify any empirical studies on in-person monitoring at railroads. However, a systematic review by Cox et al. showed that in other high-risk areas, police presence and suicide patrols have led to reductions in suicide rates [16]. Bystander presence, including surveillance units at stations, has also been associated with suicide reductions [14,61]. In a paper evaluating potential measures along Finnish railways, gatekeeper training received the highest priority, and patrols received medium priority as favorable prevention strategies, indicating expert consensus on their efficacy [54].

In the US, California and Nevada have documented in-person monitoring systems. Palo Alto had previously implemented Track Watch, where volunteers monitored crossings and security firms patrolled crossings. Similarly, the Washoe County Sheriff’s Office in Reno recruited civilian volunteers for hands-on training and monitoring nearby tracks. However, the cost and logistical challenges of sustaining these programs can be significant, as they require continuous funding for personnel wages, training programs, and equipment, as well as administrative costs for coordination and volunteer support services [55].

##### Drone Monitoring

Our review did not identify any empirical studies on drone monitoring; however, drone monitoring was identified in a report by Gabree et al. Specifically, two US initiatives through Tri Rail in Florida and the Brunswick Police Department in Maine have recently implemented drone monitoring systems. Data on their effectiveness remain limited, with reported challenges in operation costs and licensing requirements [55].

#### 3.2.3. Signage and Education Programs

##### Signage-Based Campaigns

Sign-based campaigns are integral components of railway safety initiatives aimed at raising awareness, educating the public, and promoting safe behaviors.

In Victoria, Australia, and Denmark, the use of signs at stations showed variable effectiveness [14,16,53]. Ten stations (eight metropolitan, two regional) in Victoria implemented the “Pause. Call. Be Heard” campaign, displaying signs and digital billboards with resources. An anonymous survey revealed 25% of commuters found materials moderately noticeable and 50% correctly identified the campaign’s intentions. Eighty percent reported increased help-seeking intentions, and of those, half actually engaged in help-seeking or self-care behaviors. There was a significant increase in overall Lifeline crisis calls but no significant change in suicide-related calls or rail suicidal behaviors [31].

Similarly, Valby station in Denmark implemented signs displaying suicide prevention helpline numbers, physical barriers at platform ends, and motion-sensitive lights. The station witnessed a significant reduction in suicides, although the contribution of each intervention remains unclear. Signs at the station were mentioned in 14 helpline calls, some of which involved callers deemed to be at high risk of suicide [28]. However, the authors noted that some bystanders found the messaging to be triggering, highlighting the importance of messaging that is in accordance with best practices [28]. At least fourteen US train companies have partnered with the National Suicide Prevention Helpline to advertise the helpline number at stations and on trains. Despite widespread implementation, empirical data on the effectiveness of national and local resource advertising are lacking, complicating efforts to assess usage frequency [55].

These studies suggest that signage can have some effect on the likelihood of help seeking; however, a study by Ahmed et al. that surveyed (*N* = 1011) a national sample of adults comparing likeliness to cross tracks for a variety of pedestrian-railroad scenarios using six distinct signs found that information-only signs resulted in the highest likelihood of participants crossing the railroad when no train was present (73.7% and 72.5%). For action-conveying signs, this likelihood decreased (65.6% and 33.9%), and it was further reduced for emotionally motivated signs (55.7% and 57.7%). The findings revealed that action-conveying and emotionally motivated signs were more effective in dissuading railroad crossing in high-risk situations, such as when a train was present, when crossing gates were down, or when warning lights were flashing [52]. These results suggest that more research on the types and wording and presentation of signage is needed in order to fully understand their preventative impact.

##### Gatekeeper Training

Evidence regarding the effectiveness of gatekeeper training is mixed and limited [14]. While some studies suggest a reduction in suicides at hotspots, insufficient empirical evidence exists to confirm their efficacy. Furthermore, most studies are non-specific to railway settings [14,16,62,63].

A study by Katsampa et al. conducted semi-structured interviews with individuals who intervened when someone appeared suicidal at stations or tracks. Participants commonly reported a lack of confidence and training as a barrier to intervening [27]. Increased confidence, knowledge of warning signs and positive attitudes have been reported following gatekeeper training [17,64].

##### Media Reporting

Media coverage of railway suicides can significantly influence public awareness, risk perception, and response to safety measures. Safe media reporting has been shown to improve suicide rates following an event through the Papageno effect [14,16,53,65]. Conversely, irresponsible reporting can exacerbate suicidal behavior, known as the Werther effect [14,15,49,66].

Several studies examined the role of reporting following the suicide of Robert Enke, a prominent German footballer, in 2009. Significant increases in railway suicides of between 44 and 81% (depending on the index period evaluated) were found in the immediate months following Enke’s death [47,48,49]. One paper noted a 18.8% net increase that persisted in the 2-year period following Enke’s death. There was no significant increase observed on the anniversaries of Enke’s suicide [46].

Implementing safe media reporting guidelines has shown promising results in reducing suicides. In mid-1987, the Austrian Association for Suicide Prevention developed media guidelines for reporting on suicides. The association held a press campaign with media outlets, educating them about appropriate reporting techniques. There was a significant reduction of 81 suicides annually following training, particularly in the high-impact media market. A strong correlation was found between the use of “suicide” and “self-murder” in headlines and the timing of suicides [39]. Several studies compared changes in suicide rates at railways specifically, with one study finding an 84.2% decrease from 19 to 3 suicides between the first and last half of 1987 and another study showing a 5-year sustained decrease by 75% after changes [32,42]. This decrease has shown to be sustained in following years [32,39,42].

A recent study by Sorensen et al. (2022) analyzed 220 media articles following the suicides of high-profile figures Anthony Bourdain and Kate Spade in the U.S., identifying an increase in imitative behaviors in response to media coverage. Though not directly focused on rail settings, Sorensen et al. (2022) provides the most recent insights into media reporting effects on suicide behavior and informed the development of the Tool for Evaluating Media Portrayals of Suicide (TEMPOS). This tool includes recommendations for journalists on how to responsibly cover suicide to reduce stigma and avoid sensationalism [65].

In the USA, Caltrain has decided to cease reporting on suicide [67].

#### 3.2.4. Other Interventions

##### Blue LED Lights

Three empirical studies examined the impact of blue LED lights installed at platform edges in 11 of 71 metropolitan railway stations in Japan in 2008. Two studies with different index periods by Matsubayashi et al. reported 75% and 84% decreases in total suicides at intervention stations [29,33]. However, subsequent research by Ichikawa et al. raised questions about the original studies’ conclusions. They argued that blue lights should not have an effect at night, when the blue lights were on and also only at the platform edges, not the middle of the platform since the middle of the platform would not be illuminated by the blue lights. Consequently, in their follow up analysis, they isolated nighttime suicide attempts at platform edges, where blue lights were placed. Upon reanalysis, there was no significant change in the proportion of nighttime attempts at platform ends after 2008 when blue lights were deployed [38]. In addition, further analysis of nighttime suicides did not decline after the blue lights were fully installed, suggesting that the illumination from the blue lights has had only a minimal or no effect.

##### Lighting

Limited research exists regarding lighting as a means of suicide prevention [6,17,68]. At Valby Station in Denmark, where a multifaceted approach including the installation of motion-sensitive lights was implemented, there was a decrease in suicide rates. However, the specific contribution of lighting remains unclear [28].

### 3.3. Risk of Bias in Studies

In assessing the risk of bias in the included studies, we considered several factors, particularly the design limitations inherent to observational studies. Many studies reported confounding variables, such as socioeconomic factors and naturalistic differences between sites, which were not consistently controlled for across all research [13,14,15,16,17,21,22,23,24,25,26,27,28,29,30,31,32,33,34,35,36,37,38,39,40,41,42,43,44,45,46,47,48,49,50,51,52]. Specifically, the studies varied in their methodological rigor, with limitations such as:

- Study design: Several studies employed retrospective analyses, which inherently limit causal inference and may introduce recall bias.
- Sample size: Smaller sample sizes in some studies reduced the statistical power, potentially leading to inaccurate estimations of intervention effectiveness.
- Reporting bias: Some studies may have selectively reported outcomes, influencing the perceived success of the interventions implemented.

## 4. Discussion

### 4.1. Summary of Findings

The review of the existing literature paints a picture of an incomplete examination of the impact of interventions and suicide prevention efforts in the railroad industry. The present scoping review identified a range of strategies, each with varying degrees of research and effectiveness. Several approaches appear to reduce the prevalence of suicidal behaviors at tracks, including platform screen doors, fencing, and practicing safe media reporting [29,30,32,33,35,36,37,39,41,46,47,48,49,50,51]. Additionally, the installation of suicide pits appears to also decrease the lethality of suicide attempts at stations [43,44].

Physical barriers across various modalities show considerable promise in preventing suicide [35,40,50]. Recommended standards of practice suggest that fencing ranging from a minimum of 6 feet to 9.5 feet tall fencing, with winglets which are small, angled extensions on the top of the fence to prevent people from climbing over [55,56,57]. Though currently part of the recommended practice in the field, the evidence base regarding exact height is unclear. Fences less than six feet or 1800 mm seem to be less effective than those that are 9 ft tall. Additionally, concerns around financial feasibility, visual impact, and potential displacement to unfenced areas have been raised [7,35,55]. However, there is little evidence supporting displacement. There is strong support for PSD efficacy, with full-height PSDs eliminating all suicides and half-height PSDs significantly reducing suicide incidence [30,34,36,37,41].

Studies also support the importance of responsible media reporting in suicide prevention efforts at railroads. The combination of media coverage on high-profile suicides and the adoption of reporting guidelines underscores the complexity of media influence on suicidal behavior. While safe media practices have shown significant short-term reductions in railway suicides via observational studies, further longitudinal analysis is required to assess whether these effects are sustained over time or whether the impact diminishes as public attention fades. Several studies have shown that media portrayals, particularly those that sensationalize suicide or fail to include responsible reporting guidelines, can create a “copycat effect”, leading to an increase in suicides that mimic the high-profile events [46,47]. In contrast, media outlets that followed the safe reporting guidelines saw significant reductions in suicide rates [32,39,42,65]. Several of the studies evaluated noted that further research is needed to determine the long-term impact of media guidelines and establish causality, as relatively short index periods in previous studies prevent definitive conclusions.

The review of the literature shows that signage continues to be an intervention that has some potentially positive effects but does not demonstrate a reduction in suicidal behaviors or attempts. Methodological limitations such as convenience sampling, recall bias, and cross-sectional study design were found [28,31]. However, the Ahmed study suggests that the types, wording, and presentation of signage highly influence their impact on railroad crossing and trespass behavior. Signage that includes both emotional and action-based content are more likely to have an impact on potentially risky behaviors [52].

CCTV shows potential for aiding in preventing suicides, although the evidence base is more limited. CCTV offers detection of high-risk individuals effectively in small studies; however, findings are based on a small sample of pre-recorded data rather than real-time monitoring. Detection is not equivalent to intervention, however, and requires further study. As additional studies are conducted with CCTV, procedures and techniques for intervention should be described and evaluated as well. Moreover, ethical concerns regarding privacy and data usage have been raised and will need to be addressed [7,55].

Finally, initial results suggest that suicide pits decrease injury severity, but they have not been shown to deter suicide attempts and related behaviors [43,44]. Similarly, while initial evaluation of blue lights showed significant improvements to suicide rates, follow-up research, with a higher degree of methodological quality, has raised serious questions about their true efficacy and suggest that the effects of blue light are not significant [29,33,38].

Overall, safe media practices and PSDs appear to be interventions with the most robust support, though research on both is still limited to a handful of papers. Several additional interventions and strategies have been proposed including in-person monitoring, drone monitoring, sign-based campaigns, education and gatekeeper training, lighting enhancements, and train design modifications [25,27,28,31].

This review contributes to the existing body of literature by specifically focusing on prevention strategies within the context of railway suicides, an area that has received limited attention in previous studies. By synthesizing data from diverse interventions across multiple countries, this paper highlights both the effectiveness and implementation challenges of suicide prevention, offering a comprehensive overview that enhances our understanding of how these measures can be optimized.

### 4.2. Displacement Effects

Several studies analyzed displacement effects when evaluating the effectiveness of suicide prevention interventions at railroads. Displacement occurs when the implementation of an intervention leads to a reduction in suicidal behaviors at one location but inadvertently shifts these behaviors to other nearby locations. Failure to account for displacement can result in incomplete assessments of intervention efficacy and potentially mask the true impact of the intervention.

This review found that interventions such as fencing showed varied degrees of success in mitigating displacement effects. Fredin-Knutzén and colleagues reported a decrease in suicides after the installation of 1 m of mid-track fencing at an intervention station but an increase in suicides by 162% at the other six stations along the same line. It should be noted that the study only involved a small area of fencing and small sample size of suicides [35].

Studies investigating other interventions including PSDs and blue LED lights demonstrated no evidence of displacement effects [29,30,33,34,36,37,38,41]. The lack of a direct inclusion of measures of displacement suggests a need for a more systematic assessment across future investigations. However, by not merely relocating suicidal behaviors but actively preventing them, these interventions offer promise in reducing the overall incidence of railway suicides and promoting safety across railway networks.

### 4.3. Relationship to Previous Literature

Previous systematic reviews and theoretical evaluations underscore the efficacy of physical barriers and media reporting. In particular, Barker et al. examined four interventions—PSDs, blue lights, suicide pits, and media reporting—and concluded that PSDs have the strongest support, particularly when they are full-sized [15]. Similarly, another theoretical review used an expert consensus-based approach to evaluate 38 suicide and trespassing prevention strategies across 14 criteria. The most favorable evaluated interventions were awareness/educational strategies, including changes to media reporting and physical barriers like PSDs and fencing [53]. Cox et al. assessed interventions at hotspots and found that physical barriers had the strongest support. They too noted limited research scope and quality for most interventions [16]. Lastly, Havarneau identified 139 publications related to railway suicides and trespassing. Among over 100 evaluated interventions, physical barriers like fencing and PSDs were effective, particularly when installed at hotspots, bridges, and psychiatric hospitals. Media collaboration also demonstrated strong evidence, and similar to our findings, CCTVs, lighting, signs, and education campaigns lacked sufficient research and proof of effectiveness [17].

While previous empirical systematic reviews have offered valuable insights into prevention strategies, the present study addresses notable gaps in the literature. Prior research has been limited in both the quantity of available studies for review and the scope of study. The present scoping review fills this gap by examining all interventions, whether empirically implemented or theoretically proposed. Through systematic synthesis of available evidence regarding their impact on suicidal behaviors at railroads, the present review provides a more complete understanding of the efficacy of prevention strategies and identifies areas for further research and exploration.

### 4.4. Methodological Concerns

In addition to documenting the current activity in this area, the present review offers additional insights into methodological concerns as to how such studies should be constructed, described, and analyzed. Many of the interventions evaluated suffer from significant methodological weaknesses, such as small sample sizes and lack of robust quantitative data, thereby limiting the interpretation and generalizability of their findings. Moreover, the narrow focus of much of the research on specific populations or regions diminishes its applicability to broader contexts. Future research should focus on improved and expanded metrics used when evaluating the efficacy of interventions. In most of the studies we examined, the most common approach was to simply examine the change in incidence at a given location. However, given the problem of displacement, it would seem necessary to study a much larger area of railroad territory to assess the impact of an intervention at a small portion of the rail right of way. Using the entire number of rail-related suicides as the baseline would provide for a much more accurate measure. In addition, using control locations for comparison purposes would also bolster the ability to interpret findings. Additionally, simply comparing rates or number of incidents without considering a variety of factors such as time of year, time of day, and weather factors which would provide additional context.

A well-designed study should also include appropriate comparison groups and rail traffic density when designing an evaluation of the effects of interventions. Characteristics of the populations within proximity of the locations provide additional context. For example, interventions occurring near schools, encampments of the unhoused, or mental health facilities also provide insight. Simply aggregating areas may mask important risk factors. Moreover, community characteristics such as regional suicide rates, unemployment and other economic considerations are also needed. Comparisons of data tracked over several years, obtained during similar months prior to and after the introduction of countermeasures, would be most helpful. Changing patterns in both train and pedestrian traffic might also play a role.

Another consideration regarding the methods in describing interventions is worth noting. Very few of the studies reviewed provided detailed information on the height and length of fencing along the train line [35]. The lack of information on these characteristics makes comparisons next to impossible. Clearly, higher fences that provide little hand holds or toe holds, with other obstructions preventing scaling, are more effective. Describing the dimensions and materials in more detail is needed.

### 4.5. Community-Based Insights

Beyond the empirical research, anecdotal community experiences provide additional insights that highlight areas for further investigation. In Palo Alto at the Caltrain, fewer suicides and attempts were reported after in-person monitoring was established. However, it has also been observed that individuals may hide and jump in the dark or when they feel they are not being watched, indicating mixed efficacy for gatekeeper interventions. These community observations underscore the complexity of suicide prevention measures and suggest that while some interventions may show promise, their effectiveness can vary depending on the context and implementation. Such anecdotal evidence points to the need for further research at community levels to identify circumstances under which specific interventions are most effective.

### 4.6. Economic and Cultural Factors

An improved understanding of economic and cultural factors that impact prevention interventions, so that future interventions can be tailored to specific railway settings and populations, is needed. Additionally, studies examining the long-term effectiveness of specific interventions and their impact on overall suicide rates are needed to provide more robust evidence. Similarly, consolidating findings across multiple cities and countries can bolster the generalizability of research outcomes. Emerging evidence suggests that artificial intelligence can play a significant role in preventing rail suicides by analyzing real-time surveillance data, enabling quicker detection of at-risk individuals and timely interventions, making it a crucial area for future study [51,59,69,70]. Finally, research investigating the potential synergistic effects of combining multiple intervention strategies could offer valuable insights into optimizing suicide prevention efforts. By prioritizing these research directions, the present scoping review can continue to advance knowledge and improve outcomes in railway suicide prevention.

### 4.7. Limitations

The present review employed rigorous inclusion criteria and systematic data synthesis methods to ensure the reliability and validity of the results. However, certain limitations are inherent. For instance, the present review may be subject to publication bias, as positive findings are more likely to be published than negative ones. Additionally, the heterogeneity of study designs and outcome measures across included studies makes it difficult to generalize findings.

## 5. Conclusions

This scoping review contributes to suicide prevention efforts by providing a comprehensive synthesis of both physical and non-physical prevention strategies employed at railways. By identifying and categorizing various prevention strategies currently implemented across different contexts, we have outlined a diverse range of approaches that aim to reduce suicide risk. These strategies include physical barriers such as fencing and PSDs as well as non-physical interventions like responsible media reporting and community-based initiatives.

Our analysis highlights the effectiveness of these prevention strategies in reducing suicide rates, attempts, and the lethality of suicidal behaviors. Notably, physical barriers, particularly full-height PSDs, demonstrate significant promise in preventing suicide incidents, and responsible media practices contribute to positive outcomes in preventing copycats and community awareness. However, we also address critical challenges and limitations associated with the implementation of prevention strategies along railroad right-of-way crossings. Concerns regarding methodology, displacement effects, community acceptance, and economic feasibility underscore the need for more rigorous study designs and a more nuanced understanding of context-specific factors that influence the success of these interventions.

Additionally, this review identifies significant gaps in the current evidence base, emphasizing areas for future research, practice, and policy development in suicide prevention. In addition, specific attention to study design, measures, comparison groups and analyses will improve the validity of conclusions and generalizability of results. By advocating for a more integrated approach of means safety strategies, this study underscores the importance of comprehensive interventions tailored to the unique needs of diverse populations.

In conclusion, by synthesizing the existing literature and exploring the multifaceted nature of prevention strategies, this scoping review provides a valuable framework for future research and policy initiatives aimed at reducing railway suicides. The findings highlight the critical need for ongoing collaboration among stakeholders, including policymakers, transportation authorities, and mental health organizations, to enhance the efficacy of suicide prevention efforts at railroads.

## Figures and Tables

**Table 1:** Summary of the 26 studies evaluating interventions related to railways.

Author(s)	Country/Rail System and Time Period	Activity/Intervention	Evaluation Measure	Key Results	Commentary
Structural Interventions and Access Limitation					
Fredin-Knutzén et al., 2024 [50]	Stockholm, 2020	PLFs were installed at one station in Stockholm in 2020	The number of PUT, train delays and trespassers was measured using CCTV-cameras and compared before and after installation at the intervention station over a total period of 29 months (using incidence rate ratio, IRR)	No PUT incident occurred at the intervention station after the installation, compared to 1.11 per year before installation (*IRR* = 0.32). Also a significant ~90% reduction in trespasses (*IRR* = 0.10) and delay minutes post installation. The effects were observed in comparison to the three control groups	Well-designed study using CCTV observations of PUT, trespass and also train delay minutes. Three control groups based on structural design and ridership characteristics constructed for comparison
Fredin-Knutzén et al., 2022 [35]	Stockholm, 2002–2021	1 m high mid-track fencing was installed at 1 of 7 stations along a single train line	The number of suicides recorded at the intervention station before and after installation of fencing compared to suicide rates at the 6 other stations	After installation, a 62.5% decline suicides rate at the intervention with control stations showing a 162% increase in suicides after the intervention	Well-designed study using number of suicides. Three control groups based on structural design and ridership characteristics constructed for comparison
Gregor et al., 2019 [40]	Australia, 2011–2018	Examines the impact of higher corridor fencing standards on reducing non-jumping railway suicides	Compares the proportion of non-jumping suicides between areas with different levels of corridor fencing, of metropolitan Sydney (fenced) and regional areas (unfenced)	A significantly higher proportion of suicides in the non-jumping category observed in unfenced regional areas (74%) compared to fenced metropolitan areas (33%). There was a higher proportion of jumping suicides in the fenced metropolitan versus unfenced regional areas	Clearly shows a positive reduction due to fencing, but authors acknowledge differences between the two conditions (controls) compared could have been due to factors other than fencing
Xing et al., 2019 [30]	Shanghai, 2008–2017	Installed three types of PSDs (full height, 1.5m high, 1.2m high) across 94 metro stations	Used multiperiod comparisons to assess changes in suicide frequency at stations with and without PSD over time	Metro suicides declined by 90.9% after installation of PSDs. Declines for each type of suicide were: 100% with full-height, 79.2% with 1.5m high, and 60.2% with 1.2m high. No substitution effects were seen at control stations. Half-height PSDs are less effective than full-height PSDs	Clearly shows the positive effect of PSD and the reduction of suicides. Investigation of substitution effects from metro to railway was conducted; while no effect obtained, the comparison groups were not exactly equivalent
Law et al., 2009 [34]	Hong Kong Railway, 1997–2007	PSDs were installed at 8 stations owned by Mass Transit Railway and all Kowloon-Canton Railway stations	Looked at the average percent change in suicide rate based on coroner’s data, factoring in several sociodemographic variables	76 total suicides were recorded over 11 years, with a 59.9% reduction in railway suicides after PSD installation. No significant sign of substitution to unsealed platforms	Clearly shows the positive effect of PSD and the reduction of suicides. Does not consider cost to benefit analysis or assess for displacement to alternate methods of harm
Law and Yip, 2011 [41]	Hong Kong, Kowloon-Canton Railway, 1997–2007	PSDs were installed at 8 stations owned by Mass Transit Railway and all Kowloon-Canton Railway stations	Used Incremental Cost-Effectiveness Ratio (ICER) to determine cost-effectiveness, including factors such as disability-adjusted life years (DALYs), potential fare revenue loss, and passengers’ waiting time lost due to railway circulation collapse	Installation cost was HKD 2 billion (USD 256.4 million). Installation of PSDs reduced fatal suicides by 78.9% and DALYs by over 75%. Service disruptions decreased by 69.4%. ICER analysis suggests PSD installation was effective from a larger societal perspective	Shows PSD installation reduces morbidity and mortality with no substitution effects. Also demonstrates cost-effectiveness of intervention accounting for DALYs, loss of fare and delays
Ueda et al., 2015 [36]	Japan, 2004–2014	PSDs were installed at 168 stations. 73.24% had 1.3m half-height PSDs and the remaining had full-height PSDs	Used a Poisson regression to model the reduction of railway suicides before and after half-height PSD installation and compare the effectiveness of half-height versus full-height PSDs	Installation of PSDs led to a significant decrease of 76% in the number of railway suicides overall. 7 suicides occurred after installation, and all were at stations with half-height PSDS. 5 of 7 occurred because individuals climbed over the doors, suggesting less effectiveness than full-height PSDs	Data support the near total effectiveness of full height PSD
Chung et al., 2016 [37]	Seoul, Seoul Metro, 2003–2012	Installed PSDs between 2005 and 2009 at 121 stations, 2 stations had 1.65m half-height PSDs and 119 had full-height PSDs	Used Poisson regression analysis to compare the rate of subway suicides before and after installation of PSDs	Installation of PSDs resulted in a significant decrease of 89% in fatal subway suicide cases. Full-height PSDs completely eliminated subway suicides, while 3 suicides occurred at the two stations with half-height PSDs	Clearly shows the positive effect of PSD and the reduction of suicides
O’Donnell and Farmer, 1994 [43]	London, London Underground, 1973–1990	“Suicide pits”, approximately 1-m-deep channels between the rails, were installed at railway stations. They were designed to increase the likelihood of survival for individuals attempting suicide by train	Compared fatality rates among railway incidents occurring at stations with and without suicide pits	Stations equipped with suicide pits had a significantly lower fatality rate (45%) compared to stations without pits (66%)	Study was not designed to assess suicide prevention due to pits. However, it demonstrates lower fatality rates overall at stations with suicide pits
Coats and Walter, 1999 [44]	London, London Underground, January 1996 to March 1997	“Suicide pits,” approximately 1-m-deep channels between the rails, were installed at railway stations. They were designed to increase the likelihood of survival for individuals attempting suicide by train	Evaluated mortality rates of individuals who fell or jumped under trains at platforms with and without a drainage pit	Mortality was 44% for platforms with a pit compared to 76% for platforms without a pit, showing a significant difference (*p* = 0.026)	Study was not designed to assess suicide prevention due to pits. However, it demonstrates lower fatality rates overall at stations with suicide pits
Detection and Monitoring Systems					
Agarwal, 2021 [51]	CCTV footage from several stations globally	Use of Image Analysis and Computer Vision techniques on real-time CCTV feed to detect behaviors indicating suicidal intentions	Investigated the sensitivity of the proposed evaluation protocol in predicting potential suicides based on eleven patterns and visual features extracted from CCTV footage at train stations.	Despite poor CCTV footage quality and short video duration, the proposed system had a high sensitivity of 92.85% in detecting potential suicide attempts. One model showing CCTV’s hold promise as an effective means restriction strategy at train tracks	Study shows one model for predicting suicides with high sensitivity at detecting potential suicides. However, findings are limited by short and poor-quality videos
Signage and Education Programs					
Katsampa et al., 2022 [27]	United Kingdom, Interviews between April 2019 and November 2019	Semi-structured interviews conducted on individuals over 16 yo who had intervened when someone was suicidal at a station or tracks to evaluate experiences	Examined the effectiveness and impact of interventions by individuals who have intervened in potential suicide situations at a station or tracks	21 individuals with a mix of suicide intervention training levels reported interventions, either from far (ex, calling staff) or more directly. Poor confidence was the greatest barrier to action. Participants stressed the importance of training, teamwork, and non-judgmental listening/small talk	Qualitative nature provides a nuanced understanding of interventions. However, potentially limited by convenience sampling, recall bias, and cross-sectional study design
Too et al., 2020 [25]	Victoria, Australia, May 2018	“Pause. Call. Be Heard.” campaign involving installation of materials like posters and digital billboards in stations	Measured through an anonymous online survey posted at 10 intervention stations, Lifeline crisis call data, and rail suicide behaviors before and after campaign at intervention sites	Survey findings showed 25% of commuters felt materials were moderately noticeable and 50% correctly identified the campaign’s intentions. 80% reported increased help-seeking intentions and 50% engaged in help-seeking or self-care behaviors. There was a significant increase in Lifeline crisis calls but not suicide-related calls during the campaign period. There was a non-significant decrease in rail suicidal behaviors	Suggests the campaign has some potentially positive effects but does not demonstrate reduction to suicidal behaviors or attempts. Potentially limited by convenience sampling, recall bias, and cross-sectional study design
Ahmed et al., 2024 [52]	United States	Evaluated the impact of six types of signs at pedestrian-railroad crossings	Conducted a survey of 1,011 participants comparing their likelihood of crossing tracks under different scenarios	Information-only signs resulted in the highest likelihood of crossing when no train was present (73.7% and 72.5%). Action-conveying signs reduced this likelihood to 65.6% and 33.9%, and emotionally motivated signs further decreased it to 55.7% and 57.7%	High-quality study that suggests the types, wording, and presentation of signage highly influences their impact on railroad crossing and trespass behavior. Does not directly evaluate suicide rates
Media Reporting					
Niederkrotenthaler and Sonneck, 2007 [39]	Austria, Vienna Subway System, 1946–2005	In mid-1987, the Austrian Association for Suicide Prevention developed media guidelines and initiated discussions with media to cease or minimize reporting on suicide	Applied ARIMA and linear regression models to assess the impact of the media guidelines on suicide numbers, focusing on overall annual suicide numbers and the incidence of Viennese subway suicides deemed newsworthy	Annually, there was a significant reduction of 81 suicides overall. Specifically, the high-impact media market experienced a decrease of 47 suicides annually, whereas no significant impact was observed in medium- and low-impact media markets. A strong correlation was found between the use of “suicide” and “self-murder” in headlines and the timing of suicides	Demonstrates a clear reduction in subway suicides following the implementation of media guidelines. Authors note limitations due to focusing on a single reporting indicator, potential bias from noncompliant newspapers, and a restricted 5-year analysis period
Sonneck et al., 1994 [32]	Austria, Vienna Subway System, 1980–1992	In mid-1987, the Austrian Association for Suicide Prevention developed media guidelines and initiated discussions with media to cease or minimize reporting on suicide	Changes in suicide and suicide attempt numbers as reported in the two largest Austrian newspapers	A significant increase in suicides was observed from 1984 to mid-1987, followed by a 5-year sustained decrease by 75% after changes in media reporting. No significant association was found between clusters of suicides and media reports	Well-designed evaluation demonstrating a significant reduction in subway suicides after implementing media guidelines, though causality cannot be confirmed without further longitudinal analysis
Etzersdorfer and Sonneck, 1998 [42]	Austria, Vienna Subway System, 1980–1996	In mid-1987, the Austrian Association for Suicide Prevention developed media guidelines and initiated discussions with media to cease or minimize reporting on suicide	Compared subway suicide rates and media reporting before and after the implementation of the media guidelines and campaign in mid-1987	84.2% decrease in subway suicides and attempts from 19 to 3 individuals between the first and second half of 1987, with a sustained low number of incidents in subsequent years. Findings suggest the effectiveness of altering media reports in preventing imitative suicidal behavior	Demonstrates a significant reduction in subway suicides after implementing media guidelines, though causality cannot be confirmed without further longitudinal analysis
Hegerl et al., 2013 [46]	Germany, 1998–2011	Examined the role of reporting and broadcast journalism on suicide rates before and after the suicide of Robert Enke, a German footballer	Directly compared the number of railway suicides before and after Enke’s suicide. Also used incidence ratios to analyze possible “anniversary effects” in following years	Railway suicides increased by 18.8% in the two years following Enke’s suicide compared to the two years before. There was no significant increase observed during the anniversaries of Enke’s suicide, suggesting short-term effects only	Demonstrates a notable increase in railway suicides following Enke’s death. Shows increases in imitative behavior may only be short-term. Notes that methodological challenges, such as isolating media influence from other factors, emphasize the need for further research
Kunrath et al., 2011 [48]	Germany, 2004–2007	Examined the role of reporting and broadcast journalism on suicide rates before and after the suicide of Robert Enke, a German footballer	Used a Poisson regression analysis to estimate the incidence ratios of railway suicides during the index period compared to predefined control periods, controlling for temperature and unemployment rate	Adjusted Poisson regression models showed a 44–53% daily increase in railway suicidal acts during the index period compared to control period days, even after accounting for confounders	Findings clearly link media coverage of Enke’s death to increased railway suicides, highlighting the need for strict media guidelines. Limitations include lack of exposure data and demographic analysis
Ladwig et al., 2012 [47]	Germany, 2006–2009	Examined the role of reporting and broadcast journalism on suicide rates before and after the suicide of Robert Enke, a German footballer	Used two analyses: an inter-year approach comparing the incidence of railway suicides during a predefined “index period” with identical time windows in previous years and an intra-year approach comparing the number of railway suicides 28 days before and after the incidence	Railway suicides increased, with an incidence ratio (IR) increase of 81% during the index period compared to control periods in the preceding three years. Additionally, railway suicides 28 days before and after the incidence revealed a more pronounced increase, with an IR of 2.12 and a percentage change of 117.2%	Clearly shows a significant increase in railway suicides following celebrity media coverage, suggesting a copycat effect. However, limitations such as lack of media exposure data and the inability to analyze age and sex-specific trends restrict the conclusions
Schmidtke and Hafner, 1988 [49]	Germany, Deutsche Bundesbahn, January 1, 1976 to December 31, 1984	Studied the Werther effect, wherein exposure to media depictions of suicide increases suicide rates, following a television serial depicting the railway suicide of a 19-year-old male student	Compared suicide rates before and after the broadcasting of the television serial, with a focus on age and sex groups most similar to the model portrayed in the media	Found a significant increase in railway suicides among 15- to 19-year-old males after the broadcast of the television serial, with up to a 175% increase observed during the 70-day period following the first broadcast. The effect was less pronounced in older age groups and females, and it persisted for longer periods in age groups closest in age to the model portrayed in the media	Provides strong evidence for Werther effect, particularly among those whose age and sex matched the model. Rules out referral bias or method substitution as alternative explanations, though long-term effects and method substitution were not fully explored
Other/Mixed Interventions					
Erlangsen et al., 2023 [28]	Denmark, Valby Station, 2012–2021	Implemented three key interventions (1) 12 signs saying ““Is life difficult? We are here to help”, and displaying numbers for the national suicide prevention helpline and emergency services, (2) physical barriers at the ends of platforms, and (3) 2 sets of motion-sensitive lights	Monitored calls to the Danish helpline for suicide prevention and reports of suicidal incidences at the station	No suicide deaths at the Valby Station in the 14 months post-intervention, marking a significant reduction during that period. Signs installed at the station were mentioned in 14 helpline calls, some of which involved callers deemed to be at high risk of suicide	Interventions appear to associate with reduced suicide, though methodological limitations prevent causal conclusions. Does not identify which specific interventions were effective
Baumert et al., 2011 [45]	Germany, 1998–2006	Implemented the German Railway Suicide Prevention Project, a project aimed at preventing suicidal acts on the German Railway network through several measures	Calculated railway suicide rates before and after the implementation of the project, analyzing data from the Event Database Safety, which records all person accidents on the German railway track system	The absolute number of suicidal events decreased from 1006 in 1998 to 724 in 2006. The mean suicide rate during the control years (2003–2006) was 13.9% lower compared to the index years (1998–2001), and this decline remained significant even after adjusting for the overall suicide rate	Appears to show a decrease in suicide rates following implementation of the German Railway Suicide Prevention Project. Does not identify which specific interventions were effective
Matsubayashi et al., 2013 [29]	Japan, 2000–2010	Beginning in 2008, blue LED lights were installed at the platform edges of 11 of 71 metropolitan railway stations and turned on from sunset to sunrise daily	Examined changes in suicide rates before and after installation at the 11 stations with blue lights and examined differences in suicide rates between the 11 stations with blue lights compared to 60 nearby stations without blue lights	No suicides occurred at blue light stations while the lights were on; one suicide occurred at one blue light station during daytime when lights were off. Regression analysis showed an 84% (CI:14–97%) decrease in total suicides at blue light stations after installation	The study’s finding of reduced suicides at stations with blue lights does not control for potential differences between suicides occurring at the end of platforms where the lights are installed and those occurring at other parts, making it challenging to attribute the reduction solely to the blue light installation
Matsubayashi et al., 2014 [33]	Japan, 2000–2013	Beginning in 2008, blue LED lights were installed at the platform edges of 11 of 71 metropolitan railway stations and turned on from sunset to sunrise daily	Evaluated substitution effects by comparing suicide rates at 14 intervention stations before and after installation and at 5 neighboring stations along the same line	Stations with blue lights installed showed a decrease in average suicides by 74% with no systematic increase in the number of suicides at neighboring stations	The study’s finding of reduced suicides at stations with blue lights does not control for potential differences between suicides occurring at the end of platforms where the lights are installed and those occurring at other parts, making it challenging to attribute the reduction solely to the blue light installation
Ichikawa et al., 2014 [38]	Japan, April 2002 to March 2012	Beginning in 2008, blue LED lights were installed at the platform edges of 11 of 71 metropolitan railway stations and turned on from sunset to sunrise daily	Evaluated data from the Japanese Ministry of Land, Infrastructure, Transport, and Tourism to analyze suicide cases categorized as “between stations” or “at station premises”. The study focused on cases during nighttime (6pm to 5:59am) and excluded attempts within the train or involving jumping out of the train	Of the 5841 suicide attempts recorded, 43% occurred at station premises, 43% occurred at night, and 14% fell into both categories. The proportion of nighttime attempts was higher between stations (52%) compared to within stations (32%). Assuming unidentified attempts occurred at ends, 82% of attempts were at ends, with 28% occurring at night. There was no observed change in the proportion of nighttime attempts at platform ends after 2008 when blue lights were deployed, suggesting a smaller overall effect of blue lights on railway suicide prevention than originally anticipated	High-quality study with adequate control measures and refined data analysis. Results suggest blue lights have limited or no effect

## Data Availability

The data presented in this study are derived from publicly available literature and datasets. They are listed in the references section of this manuscript. Additional information on the inclusion criteria and analysis framework is available upon request.
